# NF kappaB expression increases and CFTR and MUC1 expression decreases in the endometrium of infertile patients with hydrosalpinx: a comparative study

**DOI:** 10.1186/1477-7827-10-86

**Published:** 2012-10-15

**Authors:** Yong Song, Qiushi Wang, Wei Huang, Li Xiao, Licong Shen, Wenming Xu

**Affiliations:** 1 Department of Gynecology and Obstetrics, West China Second University Hospital of Sichuan University, Sichuan, China; 2 The Chinese University of Hong Kong Joint Laboratory for Reproductive Medicine, Key Laboratory of Obstetric, Gynecologic and Pediatric Diseases and Birth Defects of Ministry of Education, West China Second University Hospital of Sichuan University, Sichuan, China

**Keywords:** Cystic fibrosis transmembrane conductance regulator, Endometrium, Hydrosalpinx, Mucin-1, Nuclear factor kappa B

## Abstract

**Background:**

Hydrosalpinx are associated with infertility, due to reduced rates of implantation and increased abortion rates. The aims of this study were to investigate the expression of cystic fibrosis transmembrane conductance regulator (CFTR), nuclear factor kappa B (NF KappaB) and mucin-1 (MUC-1), and analyze the correlation between the expression of CFTR and NF KappaB or MUC1, in the endometrium of infertile women with and without hydrosalpinx.

**Methods:**

Thirty-one infertile women with laparoscopy-confirmed unilateral or bilateral hydrosalpinx and 20 infertile women without hydrosalpinx or pelvic inflammatory disease (control group) were recruited. Endometrial biopsy samples were collected and the expression of CFTR, NF KappaB and MUC1 were analyzed using immunohistochemistry and quantitative real-time PCR.

**Results:**

CFTR, NF KappaB and MUC1 mRNA and protein expression tended to increase in the secretory phase compared to the proliferative phase in both groups; however, these differences were not significantly different. The endometrium of infertile patients with hydrosalpinx had significantly higher NF KappaB mRNA and protein expression, and significantly lower CFTR and MUC1 mRNA and protein expression, compared to control infertile patients. A positive correlation was observed between *CFTR* and *MUC1* mRNA expression (r = 0.65, *P* < 0.05); a negative correlation was observed between *CFTR* mRNA and *NF KappaB* mRNA expression (r = −0.59, *P* < 0.05).

**Conclusions:**

Increased NF KappaB expression and decreased CFTR and MUC1 expression in the endometrium of infertile patients with hydrosalpinx reinforce the involvement of a molecular mechanism in the regulation of endometrial receptivity.

## Background

Hydrosalpinx is a common disorder characterized by a distally-blocked, dilated, fluid-filled fallopian tube. The rate of occurrence ranges from 10% to 13% when diagnosed by ultrasound, and up to 30% when diagnosed by hysterosalpingography, laparoscopy or laparotomy [1]. Evidence from two meta-analyses suggests that hydrosalpinges are associated with reduced rates of implantation and pregnancy, and increased abortion rates [2,3]. Laparoscopic salpingectomy of hydrosalpinges prior to *in vitro* fertilization (IVF) can improve the odds of clinical pregnancy and ongoing pregnancy in women undergoing IVF [4]. However, the exact mechanisms which lead to reduced fertility in women with hydrosalpinges are unclear: various theories have been proposed, including increased uterine peristalsis, the mechanical barrier created by intrauterine accumulation of refluxed fluid, the embryotoxicity of hydrosalpinx fluid or altered endometrial receptivity [5].

The cystic fibrosis transmembrane conductance regulator (CFTR) is a Cl_2_ channel expressed in the epithelial cells of a wide variety of tissues, including the human endometrium. CFTR is regulated by cAMP-dependent phosphorylation, and its expression in the human endometrium has been reported to be regulated by sex-hormones such as estrogen and progestogen [6]. As an anion channel, CFTR may play a key role in the active secretion of electrolytes and fluid by the epithelial cells of the endometrium, thus providing the optimal uterine environment necessary for blastocyst implantation. Recently, CFTR has been reported to be involved in the regulation of other proteins in some human cells, including nuclear factor kappa B (NF KappaB) and mucin-1 (MUC1) [7,8]. NF KappaB is a large family of transcription factors, whose expression in the human endometrium varies throughout the menstrual cycle [9]. MUC1 is a highly glycosylated macromolecule expressed on the cell surface which is an important endometrial receptivity factor [10].

Although the patterns of expression of CFTR and NF KappaB have been reported in the endometrium, the role of these factors in the pathogenesis of hydrosalpinx and the relationship between these factors, hydrosalpinx and infertility are poorly characterized. Therefore, we examined the expression of CFTR, NF KappaB and MUC1 in endometrial tissues collected from infertile patients with or without hydrosalpinx, and analyzed the relationship between CFTR and NF KappaB or MUC1 expression in the endometrium tissues of infertile patients with hydrosalpinges.

## Methods

### Study population

The study was approved by the Medical Research Review Board of West China Second University Hospital of Sichuan University, and written informed consent was obtained from all of the patients included in the study. From September 2010 to April 2011, 51 infertile women were recruited. All patients underwent comprehensive fertility investigations, including hormone analysis, documentation of ovulation, transvaginal ultrasound, hysterosalpingography, laparoscopy and hysteroscopy, in order to exclude patients with other disorders that may cause infertility, such as ovarian disorders, endometriosis, adenomyosis, endometrial lesions, tumors or other pelvic inflammatory diseases. A total of 31/51 women were diagnosed with hydrosalpinx by hysterosalpingography followed by laparoscopic confirmation, 20/51 patients without hydrosalpinx were included as controls.

All of the patients were aged between 20 and 40 years, with regular menstrual cycles, no history or presence of any autoimmune disease, and had not received hormonal treatment during the three months before surgery (at least). There were no significant differences in the mean age, duration of infertility and body mass index (BMI) of the two groups (Table 1). The endometrial tissues were obtained from the uterine cavity during surgery. The tissues were divided into three pieces, one piece was immediately fixed in 10% buffered formalin and paraffin embedded for immunohistochemistry, one was placed into an microfuge tube and stored at −80°C for PCR analysis, and the other was fixed in 4% formaldehyde, paraffin embedded, serial 4 mm sections were prepared, stained with hematoxylin and eosin (H&E), and subjected to routine histological examination.

**Table 1:** Clinical information of the infertile patients with or without hydrosalpinx

	**Hydrosalpinx group**	**Control group**	**P values**
*Patients(n)*	31	20	ns
*Age (yrs, range)*	29.21±4.79(23–39)	30.19±5.81(22–40)	ns
*Infertility duration (yrs)*	4.80±3.68	5.00±2.35	ns
*BMI*	20.95±3.57	20.06±2.76	ns

The menstrual phase was based on the last menstrual date, followed by histological confirmation, according to the criteria of Noyes et al. [11]. In the hydrosalpinx group, 20 patients were in the proliferative phase of the menstrual cycle and 11 in the secretory phase; whereas 12 women in the control group were in the proliferative phase and 8 in the secretory phase.

### RNA isolation, cDNA synthesis and real-time PCR

Total RNA was extracted using TRIzol reagent, according to the manufacturer’s protocol (Life Technologies Inc., Carlsbad, CA, USA); the nucleotide: protein ratios (A260:A280) of all samples were within the acceptable boundaries of 1.8 and 2.1. First strand complementary DNA (cDNA) synthesis was performed using the PrimeScript RT reagent kit (TaKaRa Biotechnology, Dalian, China) following the manufacturer’s protocol at 37°C for 30 min followed by deactivation at 85°C for 8 sec. Polymerase chain reaction (PCR) was performed using the following primers synthesized by Sangon Biotech, Shanghai, China: *GADPH*, forward 5^′^-TGCACCACCAACTGCTTAGC-3^′^, reverse 5^′^-GGCATGGACTGTGGTGATGAG-3^′^; *CFTR*, forward 5^′^-TTCACCACCATCTCATTCT-3^′^, reverse 5^′^-TACATTCTCCATCACTACTTCT-3^′^; *MUC1*, forward 5^′^-TGCCTTGGCTGTCTGTCAGT-3^′^, reverse 5^′^-GTAGGTATCCCGGGCTGGAA-3^′^; *NF KappaB*, forward 5^′^-CTGAACCAGGGCATACCTGT-3^′^, reverse 5^′^-GAGAAGTCCATGTCCGCAAT-3^′^. Each well of the PCR plate contained 5 μl EvaGreen Supermix (Bio-Rad Laboratories, Hercules, CA, USA), 1 μl of each primer (10 μmol/L) and 3 μl cDNA. Amplification was performed over 39 cycles of 95°C for 30 s, 95°C for 5 s and 60°C for 10 s using the CFX96 Real-Time System (Bio-Rad Laboratories). All experiments were performed in triplicate. The threshold cycle values were normalized against the threshold value of human *GADPH* and the results were expressed as the mean ± SEM.

### Immunohistochemistry

Immunohistochemical staining was performed using rabbit polyclonal anti–CFTR (ACL-006; Alomone Labs, Israel), rabbit polyclonal anti-NF KappaB p65 (bs-0465R; Bioss, Beijing, China) and mouse monoclonal anti-MUC1 (sc-7313; Santa Cruz Biotechnology, CA, USA) as primary antibodies. Briefly, serial sections were prepared, mounted onto gelatin-coated slides, dried overnight at 37°C, deparaffinized in xylene and rehydrated through a graded ethanol series. In order to retrieve the epitopes, the slides were immersed in a bath of pH 6.0 citrate antigen retrieval buffer for 10 min at 120°C. After cooling, the sections were incubated with 3% H_2_O_2_ for 10 min to block endogenous peroxidase activity, blocked with 10% normal goat serum for 15–30 min, then incubated with the primary antibodies (CFTR 1:200; NF KappaB 1:300; MUC1 1:300) at 4°C overnight. The secondary biotinylated antibody and streptavidin-peroxidase conjugate were applied in accordance with the manufacturer’s instructions (Beijing Zhongshan Biotech Company, Beijing, China), staining was visualized using diaminobenzidine solution, and then the sections were counterstained with hematoxylin and mounted. Colorectal carcinoma tissues were used as a positive control for NF KappaB and MUC1; rat lung tissues were used as a positive control for CFTR. Negative controls were performed by incubating the sections with PBS instead of the primary antibodies.

Negative staining was defined as the absence of an immunoreactive signal in either the stromal or glandular cells. Positive staining was defined as immunoreactive signal in the stromal or glandular cells. The H-Score [12] was used to assess glandular and stromal cell staining intensity and was calculated using the following equation: H-Score = *Σ Pi* (*i* + *1*), where *i* is the intensity of staining (1, weak; 2, moderate; 3, strong) and *Pi* represents the percentage of cells stained (0–100%). The H-scores range from 0 to 4.

### Statistical analysis

Statistical analysis was performed using SPSS version 18.0 (SPSS, Chicago, IL, USA). The Student’s *t*-test was used to compare mRNA expression levels in the hydrosalpinx group and control group, and the number of cases in the proliferative phase and secretory phase in each group. The Mann–Whitney *U*-test was used to compare the immunohistochemical H-scores. Spearman’s correlation analysis was used to assess the correlations between *CFTR* and *NF KappaB* or *MUC1* mRNA expression. *P* values less than 0.05 were considered statistically significant.

## Results

### Expression of CFTR, NF KappaB and MUC1 in the endometrium of infertile patients with or without hydrosalpinx

**Figure 1:**
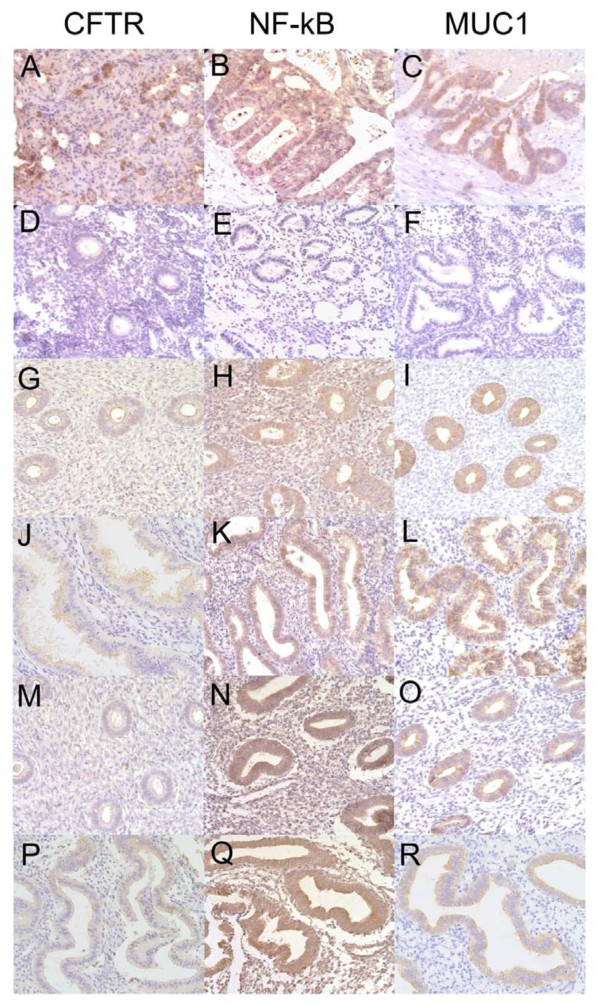
**CFTR, NF KappaB and MUC1 immunohistochemical staining in the endometrium.** Representative images of positive control CFTR, NF KappaB and MUC1 immunostaining (**A**-**C**) and negative control CFTR, NF KappaB and MUC1 immunostaining (**D**-**F**), respectively. CFTR, NF KappaB and MUC1 immunostaining in the endometrium of control infertile patients without a hydrosalpinx (**G**–**L**) and infertile patients with a hydrosalpinx (**M**–**R**). Endometrial tissues in the proliferative (**D**–**I**, **M**–**O**) and secretory (**J**–**L**, **P**–**R**) phases are shown for both groups; ×400 magnification.

As shown in Figure 1, CFTR was mainly expressed at the apical membrane of the glandular and luminal epithelial cells of the endometrium, and was almost absent in stromal cells. Noticeable expression of NF KappaB was observed in the cytoplasm of the glandular and luminal epithelial cells, with mild expression detected in the stromal cells of the endometrium. MUC1 was expressed at high levels in the membrane and cytoplasm of the glandular and luminal epithelial cells, but was not expressed in stromal cells.

Compared to the control patients, the endometrium of the infertile patients with hydrosalpinx had a significantly increased NF KappaB H-score (3.6 ± 0.5 vs. 3.0 ± 0.8; *P* = 0.03). The expression of CFTR and MUC1 in patients with hydrosalpinx were significantly lower than the control patients (2.3 ± 0.5 vs. 3.0 ± 0.7 and 2.9 ± 0.4 vs. 3.7 ± 0.5 respectively; *P* = 0.0001).

**Figure 2:**
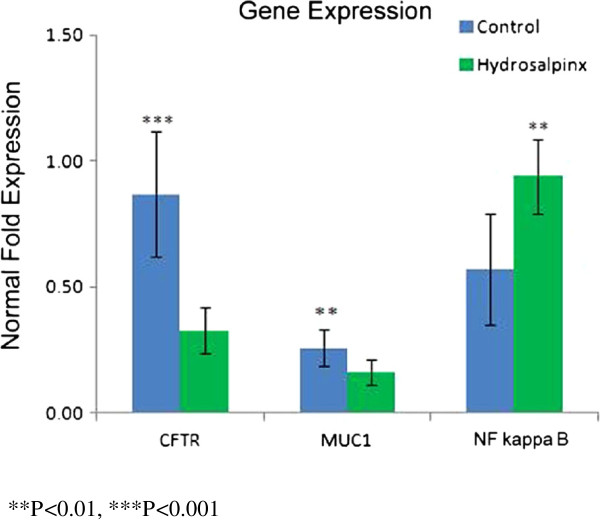
**Real-time PCR analysis of *CFTR, MUC1 *and *NF kappa B *mRNA in the endometrium with or without hydrosalpinx.***MUC1* mRNA and *CFTR* mRNA were expressed at significantly lower levels in the hydrosalpinx group than the control group, while *NF KappaB* mRNA was expressed at significantly higher levels in the hydrosalpinx group than the control group.

Quantitative real-time PCR analysis was also performed on the endometrial tissues collected from the hydrosalpinx group and the control group. In agreement with the immunohistochemistry results, *MUC1* mRNA and *CFTR* mRNA were expressed at significantly lower levels in the hydrosalpinx group than the control group, while *NF KappaB* mRNA was expressed at significantly higher levels in the hydrosalpinx group than the control group (all *P* < 0.01, Figure 2).

**Figure 3:**
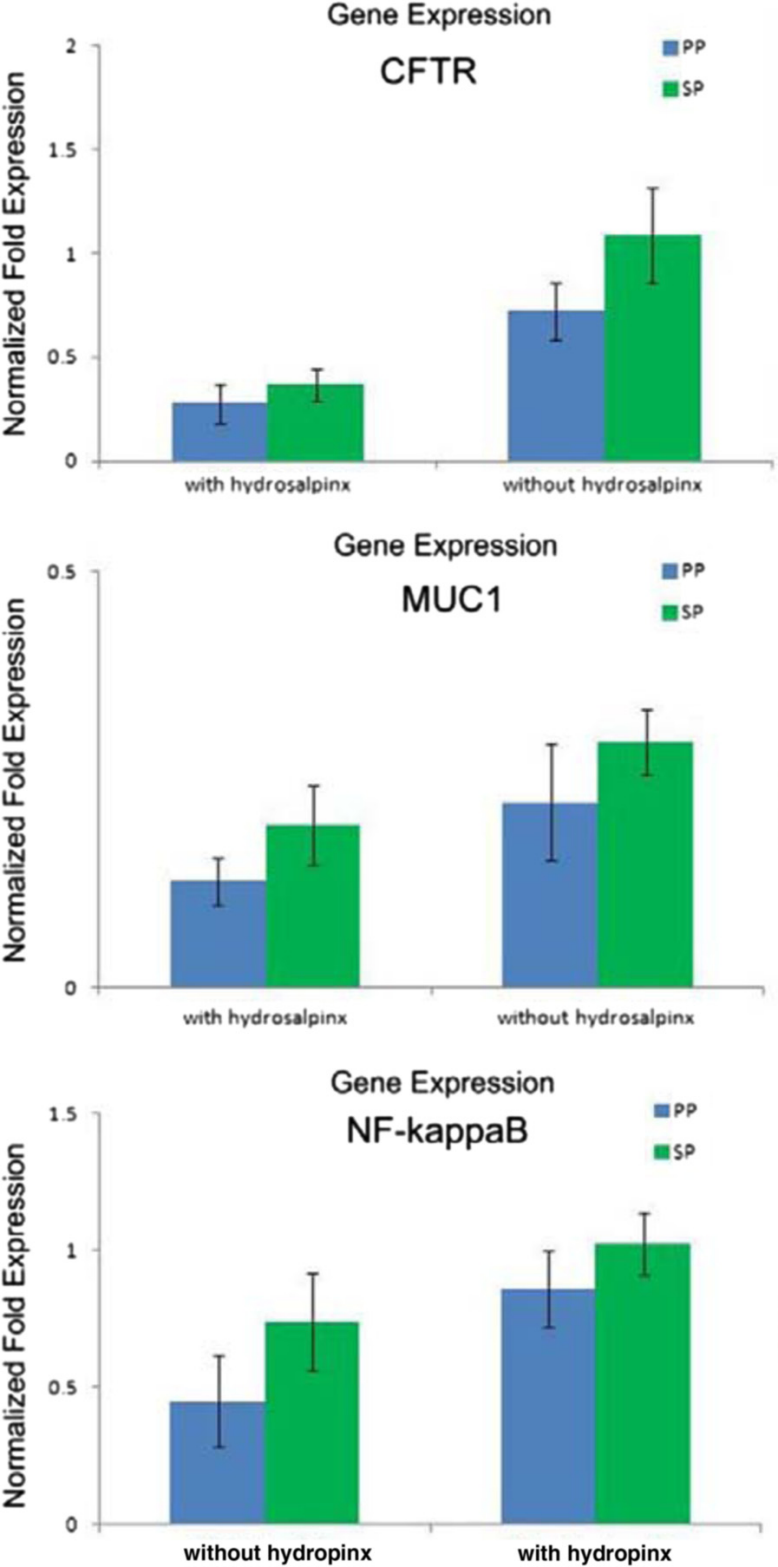
**Comparison of *CFTR, NF kappa B, *and *MUC1 *mRNA expression in endometrial tissues in the proliferative phase versus in the secretory phase of infertile women with hydrosalpinx and those without hydrosalpinx.** The expression levels of *CFTR, MUC1* and *NF kappa B* mRNA were lower in the proliferative phase and higher in the secretory phase, but these differences were not statistically significant. PP, proliferative phase; SP, the secretory phase.

A trend towards increased CFTR, NF KappaB and MUC1 H-scores were observed from the proliferative phase to the secretory phase in both groups (Table 2). Similarly, the expression levels of *CFTR, MUC1* and *NF-kB* mRNA were lower in the proliferative phase and higher in the secretory phase (Figure 3); however, these differences were not statistically significant (*P* > 0.05).

**Table 2:** CFTR, NF KappaB and MUC1 protein expression in endometrial tissues in the proliferative phase and secretory phase from infertile patients with or without hydrosalpinx

	**Hydrosalpinx group**		**Control group**		**P value**
	**PP**	**SP**	**PP**	**SP**	
*CFTR*	2.8±0.5	3.3±0.6	2.3±0.4	2.3±0.6	ns
*NFkappaB*	2.9±0.7	3.2±0.5	3.0±0.9	3.6±0.6	ns
*MUC1*	3.6±0.5	3.8±0.5	2.8±0.4	3.2±0.5	ns

All immunohistochemical staining scores are presented as H-score.

*Mann–Whitney Test. PP, proliferative phase; SP, the secretory phase.

### Relationship between the expression of CFTR, NF KappaB and MUC1 in the endometrium of infertile patients with or without hydrosalpinx

Spearman’s correlation analysis demonstrated that a positive correlation existed between the expression of *CFTR* and *MUC1* mRNA in the entire cohort of infertile patients with and without hydrosalpinx (r = 0.65, *P* < 0.05). Additionally, a negative correlation was observed between the levels of *CFTR* and *NF KappaB* mRNA expression in the entire cohort of infertile patients with and without hydrosalpinx (r = −0.59, *P* < 0.05).

## Discussion

Implantation of the embryo is an initial step in the establishment of a successful pregnancy, and involves a complex sequence of signaling events. A large number of molecular mediators have been identified and postulated to be involved in this early fetal–maternal interaction, including a variety of inter-related molecules such as adhesion molecules, cytokines, growth factors and lipids [13]. Several benign gynecological disorders, including hydrosalpinx, may hinder implantation by influencing endometrial receptivity, and salpingectomy or ligation of the salpinx before IVF can increase the success rate of IVF [4]. The presence of hydrosalpinx may affect the expression of molecules which mediate endometrial receptivity in the endometrium, such as αvβ3 integrin and leukemia inhibitory factor (LIF) [14]; however, the exact mechanisms by which a hydrosalpinx affects implantation are unclear and require further research.

CFTR plays an important role in the implantation process [6]. As an apical Cl^−^ channel, CFTR regulates Cl^−^ secretion and thus fluid volume. Additionally, CFTR is co-expressed with the epithelial NaC channel (ENaC), which has been proposed to be the major mechanism regulating uterine fluid absorption. Upregulation of ENaC and downregulation of CFTR have been observed in the endometrial epithelia of mice during preimplantation, providing a molecular basis for the ‘closure’ of the uterine lumen observed on the day of implantation. Interestingly, in this study we observed that the expression level of CFTR was significantly lower in the endometrium of infertile patients with hydrosalpinx. However, a previous study conducted in mice suggested that LPS produced by uterine infection with *C. trachomatis* induced upregulation of CFTR and abnormal fluid accumulation in the mouse uterus at diestrus [15]. There are several reasons which may lead to the inconsistencies between these studies. Firstly, different species were examined in each study. Secondly, in this study [15], the endometrial epithelia were freshly isolated from mice, and CFTR expression was analyzed only 24 hours after *C. trachomatis* LPS inoculation; therefore, it is probable that the uteri were still in the acute inflammatory phase. In contrast, the patients in our study had a history of hydrosalpinx ranging from 1 to 13 years; therefore, the disease had progressed to the chronic phase. It is possible that the decreased CFTR expression observed in infertile patients with hydrosalpinx in our study may be the result of a compensatory effect. Further studies are required to determine the expression and regulation of CFTR in the endometrium of infertile patients with hydrosalpinx.

NF KappaB is a family of transcription factors involved inflammatory and immune responses, which control cytokine and adhesion molecule gene expression in a variety of cell types, including the human endometrium. Similarly to CFTR, a non-significant trend toward increased NF KappaB mRNA and protein were observed throughout the menstrual cycle in this study. However, NF KappaB expression was mainly distributed in the cytoplasm. Nuclear expression of NF KappaB is associated with the activation of NF KappaB in pathological processes [16]. However, nuclear translocation of NF KappaB is a transient, rapid event. It is possible that the tissue sampling method or immunohistochemical techniques used in this study were not suitable for detecting subtle changes in NF KappaB activation; therefore, the lack of detectable nuclear NF KappaB expression in this study may not reflect a lack of NF KappaB activation [9].

More importantly, our study demonstrates that NF KappaB mRNA and protein expression are increased in the endometrium of infertile patients with hydrosalpinx, which has not been previously described. Hydrosalpinx is the result of chronic pelvic inflammatory disease and is characterized by fluid accumulation in the fallopian tube. NF KappaB is one of the most important regulators of pro-inflammatory gene expression, and the synthesis of a variety of cytokines, such as interleukia ( IL)-6 and LIF, are mediated by NF KappaB [17-19]. The presence of high concentrations of cytokines, including (IL)-8, tumor necrosis factor (TNF)-α and LIF, have been detected in hydrosalpingeal fluid [18]. Hydrosalpingeal fluid can flow into the uterine cavity, where the abundant cytokines may stimulate production of NF KappaB in the endometrium. In turn, increased NF KappaB expression in the endometrium may negatively influence implantation. For example, overexpression of NF KappaB in the endometrium of infertile patients with hydrosalpinx may lead to the recruitment of excessive numbers of inflammatory cells and stimulate the production of proinflammatory mediators such as IL-1, IL-6, IL-8 and TNF-α, thus leading to excessive inflammatory and immune responses in the endometrium during the peri-implantation window, which may inhibit or reduce embryo implantation. However, the exact mechanisms by which NF KappaB affects implantation in women with hydrosalpinx require further investigation.

MUC1, an important member of the mucin family, is a highly glycosylated macromolecule. When at high levels expressed on the cell surface, MUC1 can interfere with cellular adhesion via a steric hindrance phenomenon. Therefore, MUC1 may prevent adherence of the blastocyst to the endometrium by acting as an anti-adhesion molecule. The expression of MUC1 normally reduces during implantation in other species [20]; however, MUC1 is upregulated in the human endometrium during the peri-implantation period [21]. Furthermore, both MUC1 mRNA and protein expression increase several-fold from the proliferative phase to the mid-secretory phase [22]. It has been suggested that the repellent effect of MUC-1 could be of importance in guiding the blastocyst to the precise area fittest for implantation. Significantly reduced expression of MUC1 can also affect the embryo selection function of endometrium, subsequently increasing the miscarriage rate or reducing the implantation rate [23]. MUC1, an important implantation-related immune factor in the endometrium, is also expressed on the surface of T cells where it acts as an immunomodulator. In this study, we observed that MUC1 was significantly downregulated in the endometrium of infertile patients with hydrosalpinx. This result is partially in agreement with research by Li et al. [14], who reported that MUC1 expression was significantly reduced in the endometrium during the peri-implantation in patients with hydrosalpinx. Low levels of MUC1 could damage the embryo selection function of the endometrium in infertile patients with hydrosalpinx, thus increasing the miscarriage rate or reducing the implantation rate [23]. Furthermore, our study also indicated a trend towards increased MUC1 mRNA and protein expression throughout the menstrual cycle, suggesting that MUC1 is hormonally regulated during the menstrual cycle.

Interestingly, we observed a positive correlation between *CFTR* and *MUC1* mRNA and a negative correlation between *CFTR* and *NF KappaB* mRNA in the endometrium of infertile women. This indicates that CFTR can affect implantation in the endometrium not only by functioning as an anion channel, but also by acting as a molecular regulator. Previous studies have suggested a relationship between the expression of *CFTR* and *MUC1* or *NF KappaB*[7,8,24]. Kuver et al. described lower MUC1 expression in *cftr* (−/−) cells compared to wild-type cells [8]. Hunter et al. indicated that repression of NF KappaB signaling is normally mediated by CFTR (7). Moreover, Chen et al. observed a similar negative correlation between *CFTR* and *NF KappaB* expression in lung cells, and proposed that a functional negative regulation loop exists between these molecules in the lung [10]. Although the data is limited, we tentatively suggest that CFTR may positively regulate MUC1 in the endometrium, and negatively regulate NF KappaB (and possibly other factors) in the endometrium, via unknown mechanisms. It is possible that CFTR, as a chloride channel, may affect the chloride concentration in the cell, which may activate signaling pathways such as the WNK-OSR1/SPAK pathway. CFTR is also known to be either directly or indirectly involved in bicarbonate transport, which is important for pH regulation [25]. The ability of CFTR to alter pH and ion concentrations suggests that it plays a key role in the maintenance of cellular homoeostasis. Decreased CFTR expression may disrupt the cellular microenvironment and thus affect a number other factors; however, the possible role of CFTR in these processes and its ability to regulate *MUC1* and *NF KappaB* in the endometrium require further investigation.

This study has some limitations, as we did not limit patient selection to the implantation window. The precise expression levels and role of CFTR, MUC1 and NF KappaB during the implantation window in infertile patients with hydrosalpinx remain to be explored.

## Conclusions

This study demonstrates that the expression of NF KappaB is significantly increased, while the expression of CFTR and MUC1 are significantly decreased in the endometrium of infertile patients with hydrosalpinx. These results support the molecular mechanism hypothesis for the regulation of endometrial receptivity in infertile patients with hydrosalpinx. Moreover, the expression of *CFTR* mRNA in the endometrium of patients with hydrosalpinx correlates with the expression of both *NF KappaB* and *MUC1* mRNA. The specific association of these factors with hydrosalpinx-associated impaired implantation and the potential mechanisms underlying the association between CFTR and other implantation biomarkers await further investigation.

## Abbreviations

CFTR: Cystic fibrosis transmembrane conductance regulator; IL: Interleukin; IVF: In vitro fertilization; LIF: Leukemia inhibitory factor; MUC1: Mucin1; NF KappaB: Nuclear factor kappa B; TNF: Tumor necrosis factor.

## Competing interests

The authors declare that they have no competing interests.

## Authors’ contributions

YS designed the study, performed the immunochemistry and drafted the manuscript. WH designed the study and helped to draft the manuscript. QW collected the tissue samples and participated in the design of the study. LX and LS participated in the design of the study. WX helped perform experiments and reviewed/edited the manuscript. All authors read and approved the final manuscript.
